# Using Sensor Data to Identify Factors Affecting Internal Air Quality within 279 Lower Income Households in Cornwall, South West of England

**DOI:** 10.3390/ijerph20021075

**Published:** 2023-01-07

**Authors:** Christopher Johnes, Richard A. Sharpe, Tamaryn Menneer, Timothy Taylor, Penelope Nestel

**Affiliations:** 1 Faculty of Medicine, University of Southampton, University Road, Southampton SO17 1BJ, UK; 2 Wellbeing and Public Health Service, Cornwall Council, Truro TR1 3AY, UK; 3 European Centre for Environment and Human Health, College of Medicine and Health, University of Exeter, Truro TR1 3HD, UK; 4 Environment and Sustainability Institute, Penryn Campus, University of Exeter, Penryn TR10 9FE, UK

**Keywords:** indoor air quality, particulate matter, public health, housing

## Abstract

(1) Background: Poor air quality affects health and causes premature death and disease. Outdoor air quality has received significant attention, but there has been less focus on indoor air quality and what drives levels of diverse pollutants in the home, such as particulate matter, and the impact this has on health; (2) Methods: This study conducts analysis of cross-sectional data from the Smartline project. Analyses of data from 279 social housing properties with indoor sensor data were used to assess multiple factors that could impact levels of particulate matter. T-Tests and Anova tests were used to explore associations between elevated PM_2.5_ and building, household and smoking and vaping characteristics. Binary logistic regression was used to test the association between elevated particulate matter and self-reported health; (3) Results: Of the multiple potential drivers of the particulate matter investigated, smoking and vaping were significantly associated with mean PM_2.5_. Following multivariate analysis, only smoking remained significantly associated with higher mean concentrations. Properties in which <15 cigarettes/day were smoked were predicted to have PM_2.5_ concentrations 9.06 µg/m^3^ higher (95% CI 6.4, 12.82, *p* ≤ 0.001) than those in which residents were non-smokers and 11.82 µg/m^3^ higher (95% CI 7.67, 18.19, *p* ≤ 0.001) where >15 cigarettes were smoked; (4) Conclusions: A total of 25% of social housing properties in this study experienced levels of indoor PM greater than WHO guideline levels for ambient air pollution. Although there are many factors that impact air quality, in this study the main driver was smoking. This highlights the importance of targeting smoking in indoor environments in future smoking cessation and control policy and practice and of understanding how pollutants interact in the home environment. There is also a need for further research into the impact on indoor air quality of vaping, particularly due to the rise in use and uncertainty of its long-term impact.

## 1. Introduction

Air pollution causes premature death and disease. In 2012 WHO estimated that 99,000 deaths in Europe were attributable to household air pollution [1] and the recent annual report of the Chief Medical Officer for England highlighted the impact on health of indoor air pollution [2]. Indoor air pollution is a public health concern because people spend around 90% of their time indoors, with much of this being in the home environment (69%) [3,4,5,6]. This can increase individuals’ exposure to a range of diverse biological, chemical and physical agents found in the home [7], which can influence the risk of a range of allergic and non-allergic diseases [8]. Depending on the timing and extent of exposure, these can result from a complex interaction between diverse sources of outdoor pollution infiltrating the indoor environment such as road traffic [9,10,11,12], industrial processes [13] and indoor sources such as cooking/heating [14,15,16], cleaning [17,18], and environmental tobacco smoke [9,10,11,14,17,19,20,21,22,23,24,25,26]. The impact of vaping on indoor pollution is yet to be fully explored, however, several studies have measured elevated levels of particulate matter (PM) [27,28]. When indoor and outdoor pollutant sources combine, concentrations inside homes can be as much as ten times higher than outdoors [29,30]. Indoor air pollution can also disproportionately impact those in society with lower socio-economic status [31,32,33], as a result of factors such as building location and occupant density [34]. Occupant behaviour can be both a key driver of indoor pollution, and a risk factor, with low-income households spending longer in the home, due to higher levels of unemployment, whilst having higher levels of pollution-generating activities, such as smoking [33].

Reduced ventilation rates in winter months can further lead to elevated levels of PM from the sources described above [35]. Where food is burnt concentrations of PM_2.5_ up to 2000 μg/m^3^ have been recorded, which is 2281% higher than the maximum hourly mean level of 84 μg/m^3^ recorded in 2019 on Euston Road in London [15,36], although concentrations depend on indoor activities, the use of mechanical and/or natural ventilation. To put this into context, annual mean limits for ambient air pollution set by the European Union (EU) and World Health Organisation (WHO) at this time were 25 µg/m^3^ and 10 µg/m^3^, respectively, with the WHO recently halving their annual mean guideline level for PM_2.5_ to 5 µg/m^3^ [37,38]. Indoor air quality may be further impacted by some poorly designed housing interventions to make homes more affordable to heat and reduce the domestic carbon footprint (an unintended consequence). For example, inadequate heating and ventilation in more energy-efficient homes (e.g., draft proving, glazing and increased insulation) has been found to increase the risk of cardio-respiratory diseases [39]. Whole system approaches are needed to ensure more sustainable housing interventions [8] that alleviate the impact of fuel poverty on approximately 35% of homes across Europe [40] and contribute to climate change [32,41,42].

Elevated indoor particulate matter (PM_2.5_) and ultrafine particles (less than 0.1 µm) is of interest because exposure to increased concentrations has been implicated in a range of adverse health effects such as asthma [43,44]. Particulate matter can impinge on central nervous system health as they are toxic to lung and cardiovascular tissue and can enter the brain by crossing the blood-air barrier of the lungs [45]. Other health effects include low birth weight [46], the exacerbation of respiratory diseases [43,47] including inflammatory and allergic reactions [48], and increased risk of all-cause, cardiopulmonary and lung cancer mortality resulting from prolonged exposure [49]. A better understanding of factors influencing indoor PM provides an opportunity to inform future housing interventions.

Whilst the risk to health remains a public health priority, factors influencing indoor PM concentrations and their impact on health have yet to be fully explored using sensor data in homes, which is the focus of this study. Of the studies reviewed, this is the first to combine such a large dataset with monitoring over an extended period. Most studies have smaller sample sizes [11,50] or collected data over a shorter time period [10,12,17,23,26,51], or both [14,20]. A recent systematic review highlighted the relatively small number of studies that have included objective measurement of PM across large datasets [43].

In this novel study, we aim to explore the possible sources of indoor PM_2.5_ in the home environment. We will also analyse whether increases in PM_2.5_ are associated with self-reported respiratory illness. The analysis will use data from sensors recording levels of PM inside and outside the home.

## 2. Materials and Methods

### 2.1. Context

**Figure 1:**
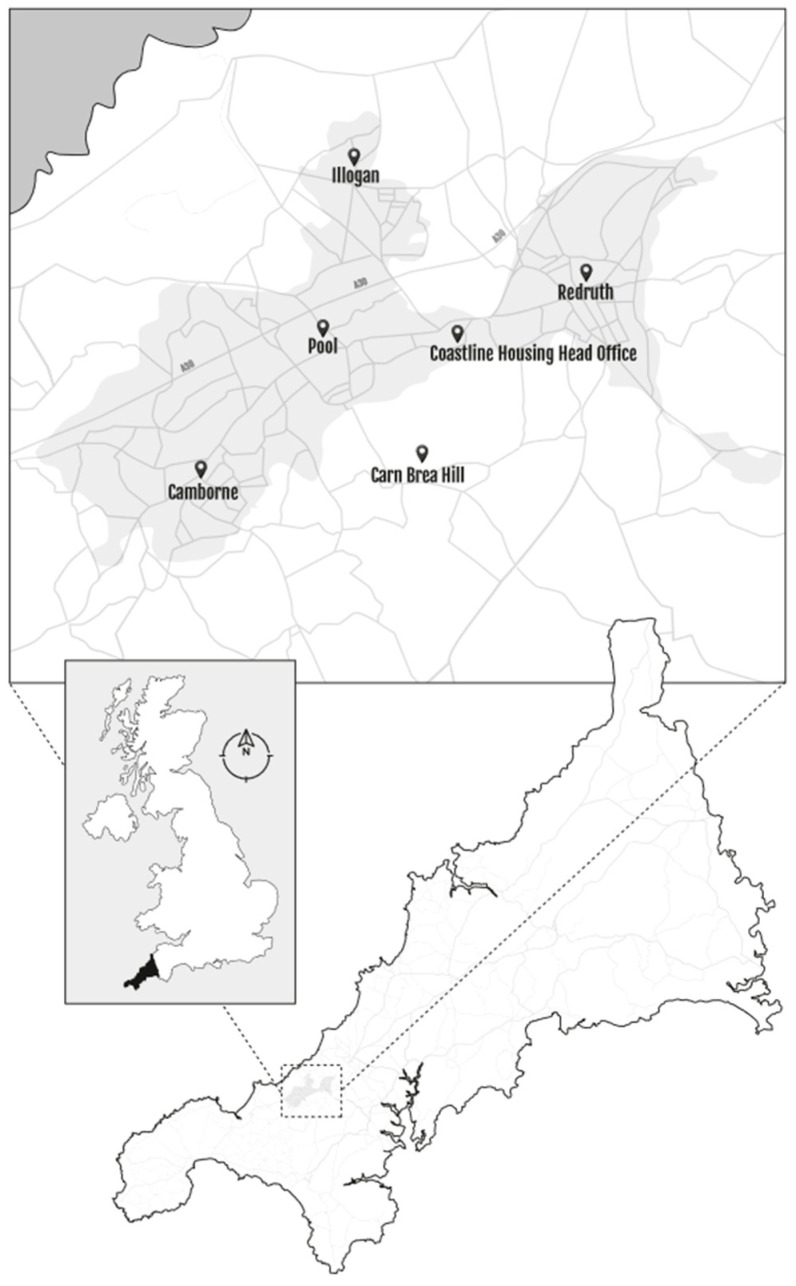
Smartline study location. Reproduced with permission from the primary author [52].

This study forms part of the large novel inter-disciplinary Smartline project (www.smartline.org.uk/) (accessed on 10 June 2020), led by the University of Exeter in partnership with Coastline Housing, Cornwall Council, Volunteer Cornwall, and the South West Academic Health Science Network. The project has recruited over 300 households in properties owned and managed by Coastline Housing with the aims of exploring the relationship between people, technology, and wellbeing in their home. The study location (Figure 1) is an area of central Cornwall in and around the towns of Camborne, Pool, Illogan and Redruth. Research activities include the use of questionnaire surveys and qualitative interviews, and the collection of sensor data on indoor and outdoor environments.

**Figure 2:**
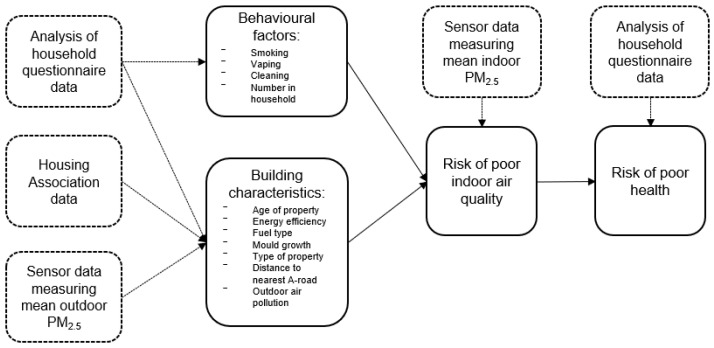
Conceptual framework of poor indoor air quality and health.

Coastline Housing is a medium-sized not-for-profit social housing association in Cornwall, South West of England. This is a largely rural area with dispersed settlement patterns including areas where there are high levels of deprivation [53]. Air quality in Cornwall is generally good, although there are towns and villages where Air Quality Managements Areas (AQMAs) have been declared as a result of high levels of NO_2_ [54]. As NOx is central to the formation of PM it is likely that PM will also be raised where there are traffic hotspots in Cornwall [55]. The conceptual framework shown in Figure 2 outlines the data sources and the possible factors that may influence poor indoor air quality, as informed by the literature.

### 2.2. Study Participants

A total of 303 households were recruited to the Smartline project in 2017–2018. This study includes results from 279 households that completed the baseline questionnaire and had air quality monitoring sensors installed and active during the period from 1 December 2018 to 30 November 2019. As previously described by Williams et al. [56], participants completed a baseline questionnaire covering self-reported household information (size, number or times vacuum/month, type of fuel used, size of any mould growth based on a 6 point scale) and health characteristics (an individual who smokes, vapes, or in the past 12 months has experienced wheeze/cough, asthma and chronic bronchitis, or emphysema/COPD and regularly lives in house). Questionnaires were completed between September 2017 and April 2018 (n = 303) with additional participants being recruited in November 2018 (n = 26). 

Study participants were compared with national and social rented sector averages to assess how representative the sample was. The percentage of female participants in this study (67.4%) is higher than the social (57.9%) and national (51%) averages from the English Housing Survey [57]. The percentage of occupiers smoking in properties in this study (37.5%) is higher than that in the social rented sector (28.6%) and nationally (14.1%) [58].

### 2.3. Indoor Sensors

Sensors were installed in 280 participants’ homes and of these, 279 were installed between October 2017 and November 2018 and were active during the study period. External sensors were installed in 53 locations in autumn 2018, which represented the geographic area where participant homes were located. The sensors used were LoRa PM_2.5_-PM_10_ Transmitters supplied by Invisible Systems (https://www.invisible-systems.com/) (accessed on 2 December 2022). These are laser-based sensors that use the light scattering method to detect and count particles in the concentration range of 0 μg/m^3^ to 1000 μg/m^3^ in a given environment. The accuracy of the sensors is ±15 μg/m^3^ at 20–30 °C. Sensor data collection has previously been described in assessments of mould risk [59]. Due to various technical issues, indoor sensor data for PM_2.5_ was available for 64% (178/279) of the properties and outdoor sensor data for PM_2.5_ for 16% (44/279) of properties. Data from both internal and external sensors was available for 30 properties. The external sensors were placed using cluster analysis [60] to provide a representative sample of external environmental measures at each home. For households without their own external sensor, values from the nearest sensor were used. To keep as close to the time of baseline questionnaire data as possible, air quality sensor data used in this study cover December 2018–November 2019. PM concentration data were recorded as PM_2.5_—measured in µg/m^3^ and a 12-month average was taken for household sensor data.

### 2.4. Behavioural Factors

Several behavioural factors have been shown to be significantly associated with indoor PM, as described in the introduction. Self-reported data on smoking, vaping, cleaning, and occupant levels were available from the household questionnaire and was included in the analysis.

### 2.5. Building Characteristics

Outdoor concentrations of PM_2.5_ were analysed for association with indoor concentrations. Distance to the nearest A-road was measured in metres from the latitude and longitude of the postal code for each home using the distance measurement tool provided in Google Maps [61,62]. Property type, construction date, and fuel type were also included in the analysis. The Standard Assessment Procedure (SAP) score [63], where 1 reflects the least energy efficiency of a property and 100 the maximum energy efficiency, was used as it may affect the air permeability of a property and therefore PM_2.5_ concentrations [64]. Building information (type, date of construction, SAP score, fuel type) was provided by Coastline Housing and linked to the participant and sensor data. Buildings, where mould is present, have been shown to have poorer indoor air quality and higher PM concentrations [65]. Household-reported data on the presence of mould was therefore also included in the analysis.

Comparing the buildings within the study sample to national and social rented averages, the distribution is broadly comparable to social rented sector averages for age, SAP score and property type (Table 1). The heating fuel type in the study properties is similar to national averages.

### 2.6. Statistical Analyses

Analyses used T-tests and ANOVAs to explore associations between mean PM_2.5_ concentrations and building, household and smoking and vaping characteristics. As indoor PM_2.5_ concentrations were positively skewed, the data were transformed using log base 10 to normalise the distribution, allowing for parametric testing. The data showed relative symmetry (skewness and kurtosis values were 0.8 and 0.2, respectively) with few large outliers that were retained as they were considered plausible. Prior to the transformation, skewness and kurtosis values were 5.6 and 45.2, respectively. Pearson and Spearman’s correlation were used where both variables were continuous. Following the analysis, the results were back-transformed to allow the accurate reporting of mean values.

Binary logistic regression was used to test the association between baseline responses for anyone in the home having self-reported wheeze/cough, asthma, or chronic bronchitis/emphysema/COPD. Variables identified as significantly associated with PM_2.5_ at the *p* < 0.05 level were included in a multivariate analysis. Backward elimination was used for the final model to create a parsimonious model by including only those variables identified as significant in the multivariate analysis. The assumptions of linearity, independence, distribution, and variance were checked following the production of the final model. Data analyses were carried out using IBM SPSS Statistics Version 26.

## 3. Results

### 3.1. Participant and Building Characteristics

A total of 279 households were included in this study (Table 2). Sixty-seven percent of the main survey participants were female and the average age was 55 (range 19–92 years). Properties were distributed equally between houses/bungalows (48.9%) and flats (51.1%), with the predominant heating fuel being gas (91.1%). The median SAP score was 73 (n = 224, LQ 69, UQ 78), which equates to a UK Energy Performance Certificate Band C. Properties were constructed between 1929 and 2018, with most (70.1%) before 1984. The median distance from the nearest A-road was 338 m (n = 257, LQ 161.5, UQ 600.5) indicating that properties were around more urban areas. 

Just over 40% (42%) of properties were occupied by a single person, 30.2% by two people, and 14.9% by 4 or more people. In over half of properties (56.2%) vacuuming took place more than twice per week. In 70.4% of households, no smoking took place inside the property. Vaping on the property was not common (14.7%). In 64.2% of households, there was neither smoking nor vaping. Among residents, 38.4% had a wheeze or cough, 28.0% had asthma and 11.5% had chronic bronchitis/emphysema/COPD.

**Figure 3:**
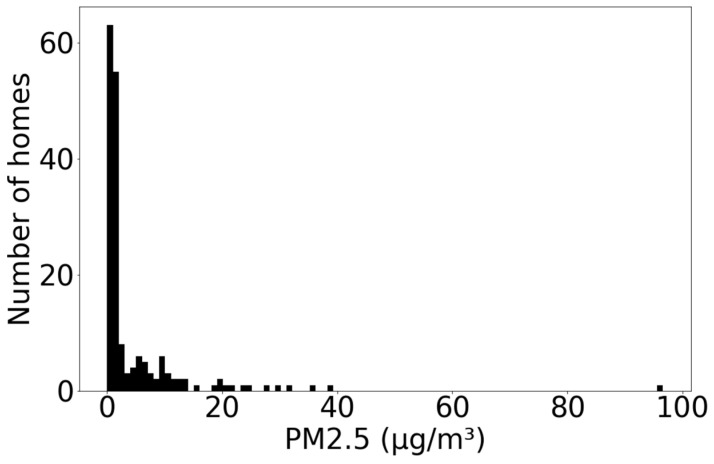
Mean annual indoor PM_2.5_ concentrations (µg/m^3^).

The annual median indoor and outdoor PM_2.5_ concentrations were 1.27 µg/m^3^ (n = 178, LQ 0.83, UQ 5.15) and 17.14 µg/m^3^ (n = 44, LQ 5.53, UQ 28.64), respectively. Figure 3 shows the distribution of indoor concentrations across the dataset. Of those properties where PM_2.5_ data were recorded, 25% had mean annual indoor PM_2.5_ concentrations exceeding the updated WHO guideline level of 5 µg/m^3^. Of the 44 properties where external sensor data were available, all had values exceeding this guideline level. The distance between the property and the nearest A-road was significantly associated with outdoor mean PM_2.5_ concentrations (*p* ≤ 0.001).

### 3.2. Association between PM_2.5_ Concentrations and Behavioural Factors

No association was found between indoor and outdoor concentrations of PM_2.5_ (Spearman’s r = 1.126, *p* = 0.893). Smoking was significantly associated with mean indoor PM_2.5_, (*p* ≤ 0.001), irrespective of the number of times smoked/day (Table 3). Mean PM_2.5_ was 1.08 µg/m^3^ (SD 1.87) in properties with no smoking compared with 9.20 µg/m^3^ (SD 2.64) for those where <15 cigarettes per day were smoked and 9.81 µg/m^3^ (SD 2.03) for those where >15 cigarettes were smoked per day. Vaping inside the house was significantly associated with mean indoor PM_2.5_ (*p* = 0.002, mean difference 2.23 µg/m^3^ (LQ 1.36 µg/m^3^, UQ 3.64 µg/m^3^)). The number of times vacuumed per month was not associated with mean indoor PM_2.5_ and no associations were found between PM_2.5_ concentrations and household size, or PM_2.5_ concentrations and mould growth.

### 3.3. Association between PM_2.5_ Concentrations and Factors Related to the Building

The distance between the property and the nearest A-road was not associated with indoor mean PM_2.5_ (*p* = 0.694)_._ Fuel group was not associated with indoor mean PM_2.5_ (*p* = 0.841). There was no association between indoor PM_2.5_ concentrations and property type (*p* = 0.083), property construction date (*p* = 0.451), and SAP value (*p* = 0.705). No association was found between concentrations and mould growth (*p* = 0.776).

### 3.4. Association between PM_2.5_ Concentrations and Factors Related to the Building

No association was observed for mean indoor PM_2.5_ and respiratory outcomes (Table 4).

### 3.5. Smoking and Vaping Analysis

The unadjusted model for both levels of smoking and vaping inside the house showed that mean indoor PM_2.5_ increased by 8.5–9 µg/m^3^ and 2.2 µg/m^3^ compared with neither taking place in the house, and these differences were significant. (Table 5). After adjusting for these variables, vaping was no longer significant while smoking significantly increased PM_2.5_ concentration. This may be due to the small sample size of 17 properties where vaping occurred without smoking. Properties in which 15 or fewer cigarettes were smoked per day predicted PM_2.5_ concentrations 9.06 µg/m^3^ higher (95% CI 6.4, 12.82, *p* ≤ 0.001) than those in which residents were non-smokers or where no smoking took place inside the home. The PM_2.5_ concentration in properties where >15 cigarettes were smoked per day was 11.82 µg/m^3^ higher than in homes without smoking (95% CI 7.67, 18.19, *p* ≤ 0.001). These findings suggest that the effect on indoor PM_2.5_ is similar irrespective of the number of cigarettes smoked. 

## 4. Discussion

In this novel study using survey and sensor data from 279 homes in Cornwall, South West of England, we found that a quarter of all properties where internal PM_2.5_ data were recorded had concentrations exceeding current WHO guideline levels for ambient air quality [37]. Of the behavioural and building characteristics analysed, we found that only indoor smoking was significantly associated with higher indoor PM_2.5_ concentration, which is consistent with existing literature [9,10,11,14,17,19,20,21,22,23,24,25]. Within this analysis, there are limitations due to the sample sizes and potential bias from self-reporting within questionnaires and the latter is worth considering in future studies. This study supports the need to further investigate the interaction between resident smoking indoors and elevated levels of PM, as well as the potential benefits of interventions that reduce smoking rates, which is a public health priority [70]. Vaping is currently viewed as a means of reducing smoking rates [71]. Whilst this study did not find an association between vaping and indoor PM_2.5_ concentration in the final multivariate model, vaping has previously been found to increase PM_2.5_ indoors [27]. It is therefore important to consider the potential harm from the inhalation of e-liquids, and unintended consequences of providing a gateway into smoking, from policies that promote vaping.

### 4.1. Factors Influencing Indoor PM

It is not clear why many of the resident and building characteristics were found not to influence indoor PM, but this is likely to be a complex interaction between lifestyles, heating and ventilation practices, which is thought to partially explain the adverse impact of some housing interventions on health and wellbeing [8]. There were several factors within the study design that may have also influenced these findings. Whilst sensors were placed in similar rooms in each of the properties, there could have been differences in their locations so results may differ. Sensors may also not have accurately picked up certain emissions due to variation in how occupiers used their properties.

Previous studies [17,18] have found an association between cleaning activities, particularly sweeping and dry dusting, and PM concentrations. It was felt that these activities disturbed PM already present in the home. Our study only considered vacuuming frequency and found no association between this activity and PM. This may indicate that vacuuming results in lower PM concentrations than sweeping or dry dusting but the lack of association may also have been due to other factors. These could include the location of sensors or short-term increases in PM not being represented in the mean levels that were analysed, which could also apply to the other findings in this study. Other indoor factors, such as the number of occupants, have been found to be associated with increased indoor PM [10,12,20,51], primarily due to an increase in household activities such as cooking. A similar association was not found in our study.

Consistent with other findings [9,10,12,13,50], this study found a significant association between the distance to the nearest A-road and outdoor PM_2.5_ concentration, but not for indoor PM_2.5_ concentrations. This may be because finer particle sizes were more likely to be found in higher quantities indoors [9] with an association between indoor and outdoor PM_2.5_ being more likely than for PM_10_ [17]. Increased levels of PM_2.5_ inside buildings may be due to finer particles being able to penetrate structures, along with indoor activities such as smoking [9]. Other studies [9,11,12] have found associations between the proximity to road and industrial sources of air pollution and indoor PM concentrations. The absence of an association between indoor and outdoor concentrations of PM_2.5_ may have been due to the properties in this study being located outside of major cities, property characteristics including air tightness and natural ventilation, or the behaviours of occupiers being different.

Existing literature was inconsistent regarding the impact on PM of building specific factors such as construction date, property type and heating fuel [10,13,19,20,50]. Our study found no association between these factors and indoor PM. Shrubsole et al. [32] found that retrofitting properties, including installation of insulation and double-glazing, could have the unintended consequence of creating poorer air quality by sealing up the property. This study found no association between SAP score and levels of PM; however, it was not possible to measure PM levels before and after any retrofit measures. With properties in this study having an average SAP score of 73, higher than the social housing average of 69 [67], it is possible that the levels of PM measured are elevated as a result of previous efforts to improve energy efficiency and reduced ventilation patterns. Mould has been found to release fine fungal particles of less than 1 µm [72]. Given this link, it was expected that homes, where mould was present, would be significantly associated with increased concentrations of PM_2.5_. It is possible that indoor sensors were not placed in areas of the home where mould was present or that the effect of other activities, such as smoking, masked any impact of mould on measuring air quality. As the presence of mould was self-reported, there is the potential for bias within responses which also may have impacted results.

### 4.2. Indoor PM and Health

No association was found between PM_2.5_ concentrations and self-reported respiratory outcomes. This finding is consistent with a recent systematic review, which reported that there is insufficient evidence using objective sensor data to better understand whether elevated indoor PM increases the risk of asthma [43]. The systematic review noted that the risk of developing or exacerbating asthma is as a result of the complex interaction between indoor air pollution and the timing and extent of environmental exposures. The focus on average annual PM values along with the cross-sectional study design may not provide sufficient detail with which to analyse this complex relationship. For example, asthma is a complex heterogenous disease, which is influenced by an interaction between genes and diverse environmental factors. These are fundamentally a population-level problem of maladapted human ecology, which need to be explored using larger sample sizes and more complex modelling [73]. Consequently, the lack of associations identified in this study could be a result of the small sample and inability to account for the interaction between multiple biological, chemical, and physical agents and health outcomes. Despite this limitation, the proportion of properties where annual mean PM_2.5_ levels exceeded guideline levels is of concern given the evidence in the literature linking levels of pollution and health [43,45,46,47,48,49].

Whilst our study did not find any association between PM_2.5_ concentrations and self-reported respiratory health outcomes, there is evidence of the link between PM and poor respiratory health [43,47]. This study highlights the impact that behavioural factors, such as smoking, can have on indoor air pollution. However, a better understanding of the interaction between indoor pollutants and ventilation rates is needed. For example, the combustible by-products resulting from environmental tobacco smoking can persist for 1.5 to 2 h before returning to normal levels [74]. The speed at which levels return to normal will be based on building factors such as air change rates and the amount of ventilation available. Air change rates will vary depending on several factors such as weather (temperature difference between inside and outside and windspeed), the building design, and how air-tight the structure is, and occupant behaviour through activities such as cooking and smoking and use of windows and mechanical ventilation systems [75,76]. Indoor air pollution therefore should be an important consideration in building design, which needs to be considered alongside diverse resident lifestyles, behaviours, cultures, and the outdoor environment. These should consider the interaction with socio-economic status, which has been found to be an indicator for increased concentrations of PM indoors [31,32]. This is important to consider because smoking prevalence is higher among disadvantaged groups [77], and those living in deprived neighbourhoods experience higher concentrations of PM when measured outdoors [78].

### 4.3. Study Strengths and Limitations

The main strength of this study is the large sample size of 279 properties with sensor data recorded over long periods, both inside and outside dwellings. To fully explore the interaction between the diverse air pollution sources and exposures, future studies will likely require significantly larger sample sizes. The use of average PM_2.5_ levels over a one-year period helps to ensure that any short-term variations in concentrations do not have a significant impact on overall results. A significant strength of this study is the collaboration between the project partners, enabling access to a large number of homes and asset management records linked to those homes, such as energy efficiency data [40].

There are several limitations to consider. The cross-sectional study design limits the possibility of evaluating causality. Self-reported questionnaires were the source of several variables and, although response rates were generally high, bias can occur as a result of the tendency for some participants not to respond to questionnaires and, within those that respond, there is a tendency for some to believe that certain factors, such as dampness and mould growth, impact health, influencing responses. The study focusses on properties owned by Coastline Housing and therefore, generalisability of findings is limited outside of the social housing sector in the South-West of England. Whilst using average annual PM_2.5_ values helped to reduce the impact of short-term variations on results, those short-term variations may also have shown subtle associations between variables and indoor air quality. 

The occupants themselves differed from national averages in terms of their characteristics, with higher rates of smoking (37.5% of households vs. 14.1% of people nationally [58]) and higher rates of asthma (28% of households vs. 13% of people nationally [66]) and COPD (11.5% of households vs. 2% of people nationally [66]). The questionnaire data were collected approximately 12 months before the data on PM were available, and changes may have occurred in household residents and variables relating to their behaviour. Several studies with smaller sample sizes required participants to complete activity diaries, thereby allowing a direct comparison of occupier activity and PM level [10,14,17]. This was not possible within this study therefore it was not possible to consider the associations between specific household activities such as vacuuming and cooking, and PM_2.5_ concentrations. All variables had missing data, including PM_2.5_ concentrations in one-third of properties, and this may have affected the results. Where data was not available, it was marked as missing in SPSS and analysis was limited to those properties where complete data was available. Data were not available when properties were unoccupied so the effect of a property being vacant on PM_2.5_ concentrations could not be assessed. Further analysis of other sources of PM_2.5_ (both external and internal) could be conducted, but this was not possible in the current study. Health data was restricted to binary responses on the presence of conditions at a single point in time, restricting the level of analysis. It would be useful to have more robust measures of health, such as asthma surveys or bronchodilator use, which could be linked to indoor air over time.

## 5. Conclusions

Building on an innovative, mixed-method study involving self-reported health and indoor air quality sensors, this study highlighted that smoking behaviour is related to increases in PM inside the home. It has been shown that 25% of social housing properties in the sample experienced levels of indoor PM greater than WHO guidelines for ambient air pollution. It highlights the importance of targeting smoking in indoor environments in future policy and practice, as well as the need for further research to better understand the interaction between smoking, vaping, and indoor air pollution. Models of higher complexity and larger sample size are needed to explore the diverse interactions between resident and building characteristics, resultant fluctuations in indoor air pollution and impact on health and wellbeing. It is also important that future studies analyse how different pollutants interact within specific environments, such as the home. Further work is also needed to consider indoor air pollution levels across different housing sectors, such as the owner-occupied and privately rented sectors and to consider different locations, particularly those with more heterogeneous outdoor air pollution. Given the climactic factors affecting air quality, further research is also needed in different regions of the world and contrasting rural and urban environments to better understand the drivers of poor indoor air quality. Increasing our understanding of factors driving indoor air pollution and resultant health outcomes will help inform future building design and housing retrofit interventions to avoid the potential unintended consequences resulting from inadequate heating and ventilation. A whole system approach is needed in the design of appropriate interventions—considering how humans interact with housing (considering ventilation, smoking, how much time is spent indoors), and the wider determinants of indoor and external environments.

## Figures and Tables

**Table 1:** Comparison of study participant and building characteristics with national and social rented sector averages [57,58,66,67,68,69].

Variable	Study Participants	Social Rented Sector	National
Age (Years)	55	53	54
Gender (Female)	67.4	57.9	51
Smokers (%)	37.51 ^1^	28.6	14.1
SAP score	73.21	69	63
Property type (%)			
House/Bungalow	48.9	56.2	79.9
Flat	51.1	43.8	20.1
Fuel type (%)			
Gas	91.1		85.8
Other	8.9		14.2

^1^ Results at household, not individual level.

**Table 2:** Baseline characteristics of participants and their properties (n = 279).

Variable	n	Mean (Range)	n (%)
Gender of main participant	258		
Male			84 (32.6)
Female			174 (67.4)
Age of main participant	258	55 (19–92)	
Property type	272		
House/Bungalow			133 (48.9)
Flat			139 (51.1)
Fuel type	271		
Gas			247 (91.1)
Other			24 (8.9)
SAP score	224	73 (26–86)	
Property construction date	271		
Pre 1965			115 (42.4)
1965–1983			75 (27.7)
1984–2001			31 (11.4)
2002 onwards			50 (18.5)
Number in household	262		
1			110 (42.0)
2			79 (30.2)
3			34 (13.0)
4 or more			39 (14.9)
Number of times vacuumed per month	258		
Less than once/week			65 (25.2)
Between once and twice/week			48 (18.6)
More than twice/week but less than once/day			51 (19.8)
Once/day or more			94 (36.4)
Level of smoking inside house	260		
None			183 (70.4)
≤15 times per day			50 (19.3)
>15 times per day			27 (10.4)
Vaping in property	279		
Yes			41 (14.7)
No			238 (85.3)
Wheeze or cough within household	279		
Yes			107 (38.4)
No			172 (61.6)
Asthma within household	279		
Yes			78 (28.0)
No			201 (72.0)
Chronic bronchitis/Emphysema/COPD within household	279		
Yes			32 (11.5)
No			247 (88.5)

**Table 3:** Results of statistical tests of association between variables and mean indoor PM_2.5_.

Variable	n	Mean Indoor PM_2.5_	
		Mean (SD)	*p*
Mean outdoor PM_2.5_	30		0.893 (coefficient −0.026) ^2^
Level of smoking inside house	165		<0.001 (f = 154.826) ^3^
None	121	1.08 (1.87)	
≤15 times per day	26	9.20 (2.64)	
>15 times per day	18	9.81 (2.03)	
Vaping in property	178		0.002 ^4^
Yes	26	3.95 (2.86)	
No	152	1.78 (3.30)	
Number of times vacuumed	164		0.154 (f = 1.774) ^3^
Less than once/week	45	1.39 (2.70)	
Between once and twice/week	34	2.21 (4.69)	
More than twice/week but less than once/day	31	1.86 (2.89)	
Once/day or more	54	2.31 (3.01)	
Number in household	165		0.186 (f = 1.624) ^3^
1	75	2.26 (3.81)	
2	52	1.57 (2.89)	
3	20	2.41 (3.07)	
4 or more	18	1.41 (2.13)	
Presence of mould	163		0.776 (f = 0.500) ^3^
None	93	2.03 (3.73)	
One or two spots	9	1.22 (2.01)	
Several small patches (postage stamp)	11	1.51 (2.55)	
Bigger than a post card	10	2.49 (2.35)	
Up to an arm’s length (1 m)	18	1.79 (2.51)	
Bigger than an arm’s length	22	1.89 (2.95)	
Distance to A road	176		0.694 (coefficient −0.030) ^1^
Fuel type	174		0.841 ^4^
Gas	162	1.98 (3.30)	
Other	12	2.13 (3.33)	
Property type	174		0.083 ^4^
House/Bungalow	75	1.66 (3.19)	
Flat	99	2.27 (3.33)	
Property construction date	174		0.451 (f = 0.884) ^3^
Pre 1965	74	1.86 (3.15)	
1965–1983	48	2.48 (2.81)	
1984–2001	23	1.96 (3.24)	
2002 onwards	29	1.64 (1.49)	
SAP score	143		0.705 (coefficient −0.032) ^1^

^1^—Pearson’s correlation. ^2^—Spearman’s correlation. ^3^—ANOVA. ^4^—Two sample *t*-test.

**Table 4:** Odds ratio for mean indoor PM_2.5_ and respiratory outcomes.

	Wheeze/Cough			Asthma			Chronic Bronchitis, Emphysema or COPD		
	n	Odds Ratio (95% CI)	*p*	n	Odds Ratio (95% CI)	*p*	n	Odds Ratio (95% CI)	*p*
**Mean Indoor PM_2.5_**	65	1.02 (0.98, 1.06)	0.350	50	1.01 (0.97, 1.04)	0.798	19	1.07 (0.95, 1.19)	0.272

**Table 5:** Results of smoking and vaping analysis.

Model Outcome: Indoor Annual Mean PM_2.5_	Unadjusted Models (From Table 3)			Adjusted Model *		
	Beta	95% CI	*p*	Beta	95% CI	*p*
**Current smoking level in house**						
None (ref)	1			1		
≤15 times per day	8.49	(6.30, 11.44)	<0.001	9.06	(6.40, 12.82)	<0.001
>15 times per day	9.05	(6.39, 12.83)	<0.001	11.82	(7.67, 18.19)	<0.001
**Vape in house**						
No (ref)	1			1		
Yes	2.22	(1.36, 3.64)	0.002	0.55	(0.29, 1.07)	0.593

* Adjusted for current level of smoking in house and vaping in house.

## Data Availability

The majority of data are available by registering at www.smartline.org.uk/data (accessed 10 June 2020).
